# Controlling Powered Prosthesis Joint Impedance Over Continuous Stance Transitions Between Walking and Stair Ascent/Descent

**DOI:** 10.1109/TNSRE.2026.3683020

**Published:** 2026

**Authors:** Ross J. Cortino, Shihao Cheng, Robert D. Gregg

**Affiliations:** Department of Robotics, University of Michigan, Ann Arbor, MI 48109 USA

**Keywords:** Prosthetics, stairs, legged locomotion, biomimetics, biomechanics, control design, impedance, optimization, steady-state, switches

## Abstract

Transition strides between level-ground walking and stairs are important parts of everyday locomotion that help maintain balance while sustaining the momentum of the user between these activities of daily living. However, most individuals with transfemoral amputations are unable to perform these continuous transitions with their conventional passive prostheses, instead being forced to initiate transitions with a specific leg or pause at the threshold of the staircase. Powered prostheses have the potential to allow for continuous transitions due to their ability to provide positive work and active control during level-ground walking and stair locomotion, but modern impedance control approaches switch discretely between steady-state controllers instead of emulating continuous joint biomechanics. This work presents a phase-based hybrid kinematic impedance controller that provides biologically-inspired knee and ankle impedance during continuous stance-phase transitions between level-ground walking and stair ascent/descent, assuming high-level knowledge of the transition stride. In an offline analysis of N=12 participants, we show that our continuous stance transition modeling approach significantly outperforms a typical discrete switching strategy in most cases. To experimentally implement the transition model, we define a common thigh-based phase variable for both steady-state and transition strides, giving the user control over prosthesis stride progression. Pilot experiments with two K4 transfemoral amputee participants using a powered knee-ankle prosthesis demonstrate biomimetic kinematic/kinetic features during stair ascent/descent transitions for two stair incline configurations, without subject-specific tuning of control parameters.

## I. Introduction

Modern knee prostheses struggle to perform biomimetic stair climbing due to the lack of net-positive work when ascending and active control of foot placement and energy dissipation when descending [1], [2], [3]. Utilizing these devices, individuals with transfemoral (TF) amputation often experience increased cognitive load and fall-related incidents, especially during transitions to/from staircases [4], [5], [6].

To mitigate these fall risks when transitioning between level-ground walking and stair locomotion, prosthesis users must employ strategies such as always initiating the transition with a specific leg and foot placement, and/or pausing at the top or the bottom of the stairs to maintain balance [1], [5]. For example, during a transition from walking to step-over-step stair ascent, the user must employ rapid hip flexion to ensure proper foot clearance. To achieve this without toe stubbing, the user must step on the level-ground surface with the intact, contralateral limb at a sufficient distance from the stair step. Similarly, during stair descent, the user is trained to step with the intact limb on the top step to help maintain balance going into stair descent with the prosthesis [7]. These strategies limit the user’s ability to transition with the prosthesis when taking the final stance stride at the bottom or top of the staircase. As a result, users often have to pause or slow down their gait to transition properly, depending on their approach [5].

Powered TF prostheses [8], [9], [10], [11], [12], [13] promise to address limitations of passive devices by performing net work with active joint control during stair locomotion [14], [15], [16], [17], [18], [19], [20], [21]. In particular, use of a passive prosthesis is associated with increased joint power, strain, and compensations [22], [23], [24]—especially during stair locomotion [25]—often leading to secondary conditions such as arthritis and back pain [26], [27], [28]. These gait abnormalities have been mitigated by providing biomimetic joint mechanics through a powered device during steady-state walking [29], [30], [31], [32], [33] and stair climbing [34], motivating their use to achieve fluid, continuous transitions between these activities.

However, state-of-the-art control strategies for powered legs fail to replicate natural biomechanics during certain walk-to-stair (W2S) and stair-to-walk (S2W) transitions due to a reliance on discrete switching. For both transition types, powered prostheses typically switch discretely between distinct impedance-based controllers tuned for steady-state stairs (S_ss_) and level walking (W_ss_) [35]. These controllers typically switch modes at foot-strike (FS), mid-stance, and toe-off (TO) events in the transition stride, which is defined as the stride beginning at prosthesis FS with both feet on the old terrain and ending at the subsequent prosthesis FS on the new terrain. In addition to these common switching points, optimal points have been investigated for impedance control of walk-ramp transitions [36] and kinematic control for walk-stair transitions [37]. While appropriately timed switching with temporal fading between controllers can facilitate smooth transitions, this paradigm cannot fully capture the differences between steady-state and transition biomechanics found in able-bodied (AB) data [6], [38], [39], [40].

**Fig. 1.**
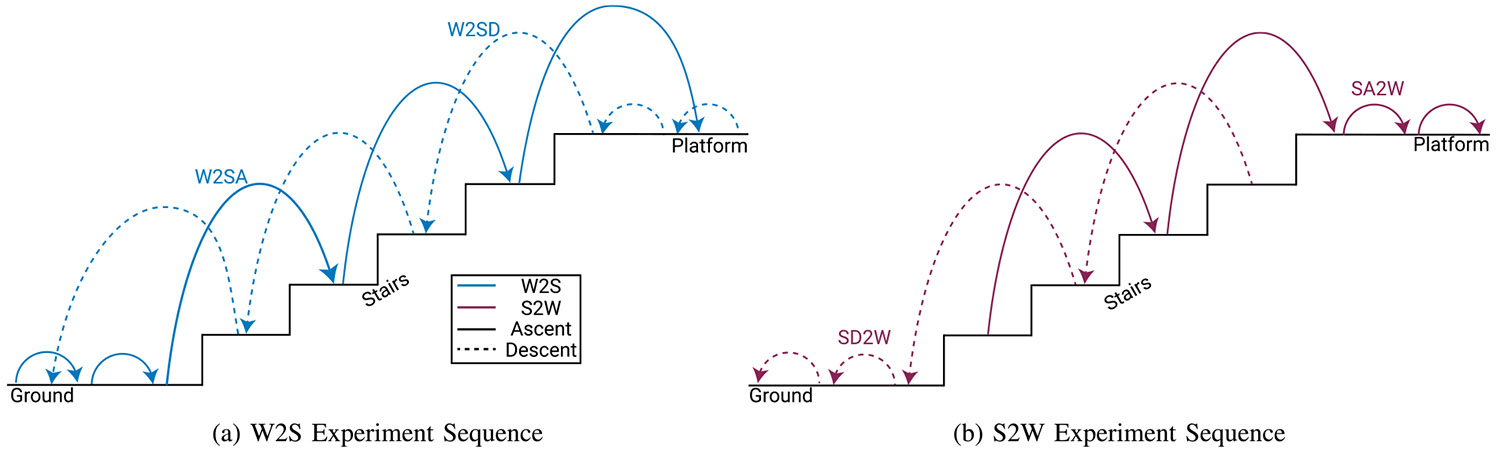
Visualization of the W2S and S2W stance transitions and the stride sequence used in the experimental protocol. The stance portion of the stride happens at the base of the arrow, and the arc of the arrow illustrates the swing portion of the stride. Stair ascent and descent transitions are denoted with A and D, respectively. During stair portions of each W2S transition trial, the participant starts two walk strides away from the staircase on either the ground for ascent or platform for descent. For stair portions of a S2W transition trial, the participant starts with a stair stride on the first step of the staircase.

Recent work improved kinematic biomimicry over stair transition strides by continuously interpolating between steady-state models of joint kinematics generated from AB data [37], but biomimetic kinetics remain elusive. This study demonstrated that biomimetic kinematics enable proper toe clearance and touch-down positioning during *swing* transitions, where the prosthesis leads the transition to new terrain requiring deviations from steady state during its swing phase [37], [38]. However, position control creates unnaturally rigid interaction between the human, robot, and environment during *stance* transitions, where the prosthesis acts as the trailing limb requiring deviations from steady state during its stance phase (Fig. 1). Joint kinetics must be considered in the controller design and validation—going beyond [37]—to ensure comfortable interaction and biomimetic energy exchange. There remains a key gap in controlling biomimetic kinetics to achieve these benefits during stance transitions.

These limitations could potentially be addressed by using impedance control during the stance phase, which has been shown to produce biomimetic joint kinetics and compliant interaction with the ground during steady-state activities [14], [16], [17], [20], [25], [41]. Impedance control regulates the relationship between joint angle and torque instead of controlling joint angle directly (which also reduces sensitivity in the phase mapping due to the small thigh range of motion during stair descent [21]). Hybrid kinematic impedance control (HKIC) uses impedance control for compliant interaction during stance and position control for predictable foot placement during swing, leveraging phase-based models generated from AB datasets in [32]. While HKIC was extended to steady-state stair ascent/descent in [21], transitions to/from walking have only been implemented via discrete switching [42], which failed to reproduce normative transition kinetics (e.g., frequently mistimed). Another hybrid control approach uses a heuristic-based impedance controller during the stance phase of level-ground walking, stair climbing, and transitions between these activities [43]. However, this hand-tuned approach was not evaluated against AB biomechanics and required compensatory strategies commonly used with passive prostheses during stair descent and associated transitions (i.e., pivoting the prosthetic foot over the edge of the stairstep). Hence, powered prosthesis users still lack the ability to perform stair transitions with both biomimetic kinematics and kinetics.

To address these limitations with state-of-the-art controllers, we propose a data-driven, phase-based HKIC approach that enables continuous, biomimetic stance transitions between level-ground walking and stair ascent/descent. This approach allows for the completion of all four stance transitions: walk-to-stair-ascent (W2SA), walk-to-stair-descent (W2SD), stair-ascent-to-walk (SA2W), and stair-descent-to-walk (SD2W). Our novel contributions include the construction of a data-driven, continuously-varying transition model that interpolates between our previously-defined W_ss_ and S_ss_ impedance models as a function of stance phase progression, similar to the interpolation approach defined for kinematic models in [39] but instead with a kinetic output. After modeling two stair inclinations, we show that an incline-independent approach reduces model complexity without reducing performance during both ascent and descent transitions. Using data from N=12 AB subjects in [38], we perform an offline comparison of our continuous stance transition impedance models to a common discrete switching strategy between our steady-state walk and stair models, demonstrating significant improvements in performance for most cases. Finally, utilizing a common phase variable definition from [21] for all activities, we show preliminary evidence that our continuously-varying impedance model can produce biomimetic joint biomechanics during continuous walk-stair stance transitions at two stair inclinations for two TF (K4) participants, with no participant-specific tuning of the impedance model or phase parameters. Combining these biomimetic stance transitions with biomimetic swing transitions from [39] (and automatic activity classification from [42]) has the potential to enable more natural and seamless multi-terrain locomotion for above-knee amputees who frequently navigate the home and community.

This work is organized in the following manner. Section II provides a high-level overview of the phase-based control approach utilized in this work, detailing the differences between the impedance control utilized in stance and the kinematic control in swing. Section III introduces the data-driven optimization approach to generating the continuously-varying transition model and compares this model to a simpler transition model that discretely switches between the appropriate steady-state model. Section IV presents the experimental evaluation of our stance transition model with two TF amputee participants, focusing on phase variable progression and joint kinematics, kinetics, and work. Section V discusses these results, participant feedback, the limitations of our approach, and potential future work. Section VI concludes this paper, summarizing key takeaways from this work.

## II. Control Architecture for Stance Transitions

The proposed HKIC controller for stance transitions utilizes our previous HKIC control approaches for variable-speed/incline walking [32] and variable-height stair ascent and descent [21]. Taking inspiration from these previous works, the task variable χ represents a continuous variation of an activity, specifically the gait speed and ground incline during walking or the staircase incline during stair ambulation. In this work, we assume walking occurs at 1 m/s and a ground incline of 0° because the reference AB data [38] does not specify a walking speed for level-ground walking. Stair incline varies based on the known incline of the staircase taken from the dataset and replicated in our experimental setup.

During stance, the controller utilizes a continuously varying impedance model that interpolates between S_ss_ and W_ss_ models, modulating stiffness, damping, and equilibrium angle as continuous functions of stance phase and task variables. This interpolation approach takes inspiration from previous continuous transition models focused on position control of powered legs [37], [39]. In swing, the controller utilizes a position controller that modulates desired joint angles as functions of swing phase and task. The proposed transition controller focuses on the stance portion of intact-leading transitions (see Fig. 1), so the swing controller is that of the steady-state activity that follows the transition. We define the stance portion of the stride as the portion between FS and TO while the foot is in contact with the ground. The rest of the stride, until the next FS, is defined as swing.

### A. Common Phase Variable Definition Across Activities

We utilize a similar phase estimation technique as shown in [21] and apply it to level-ground walking, stair ascent/descent, and transitions between these activities. This phase variable definition decouples the stance and swing portions of the gait cycle, defining them by a stance phase variable $s_{\mathrm{st}}$ and swing phase variable $s_{\mathrm{sw}}$, removing interdependence of the impedance and kinematic control frameworks on one another. The gait cycle phase variable s is defined as

(1) $$s=s_{\text{st}}\cdot {\hat{s}}^{\text{TO}}+s_{\text{sw}}\cdot (1-{\hat{s}}^{\text{TO}}),$$

where ${\hat{s}}^{\mathrm{TO}}$ denotes the average value in normalized time (based on AB data) at which TO occurs for a given activity mode ζ and task variation $\chi^{\zeta }$ (i.e., walking speed/incline or stair incline). The activity mode ζ∈{W,S,W2S,S2W} with W denoting walking, S denoting stairs, W2S denoting walk to stair transitions, and S2W denoting stair to walk transitions. The full definition of the phase variable utilized in this work can be found in [21]. The phase parameters utilized for a given $\chi^{\zeta }$ were calculated from AB thigh kinematics and the average AB phase progression based on the AB dataset [38].

### B. Stance Impedance and Swing Kinematic Controllers

During stance, we calculate joint torque $\tau_{\mathrm{st}}$ with an impedance controller where the impedance parameters vary as a function of phase and incline. For steady-state models S_ss_ and W_ss_, we input the calculated stance phase $s_{\mathrm{st}}$ and known task $\chi^{\zeta }$ to determine the joint stiffness $K^{\zeta }$, equilibrium angle $\theta_{\mathrm{eq}}^{\zeta }$, and damping $B^{\zeta }$ for our impedance torque control law

(2) $$\tau_{\text{st}}^{\zeta }=-K^{\zeta }(s_{\text{st}},\chi^{\zeta })\theta -B^{\zeta }(s_{\text{st}},\chi^{\zeta })\dot{\theta }+\delta^{\zeta }(s_{\text{st}},\chi^{\zeta }),$$

where $\delta^{\zeta }(s_{\mathrm{st}},\chi^{\zeta })=K^{\zeta }(s_{\mathrm{st}},\chi^{\zeta })\theta_{\mathrm{eq}}^{\zeta }(s_{\mathrm{st}},\chi^{\zeta })$. It is important to note that $\theta_{\mathrm{eq}}^{\zeta }$ does not necessarily match the desired kinematics during stance, enabling the control law to provide torque when following the ideal kinematics [44].

**Fig. 2.**
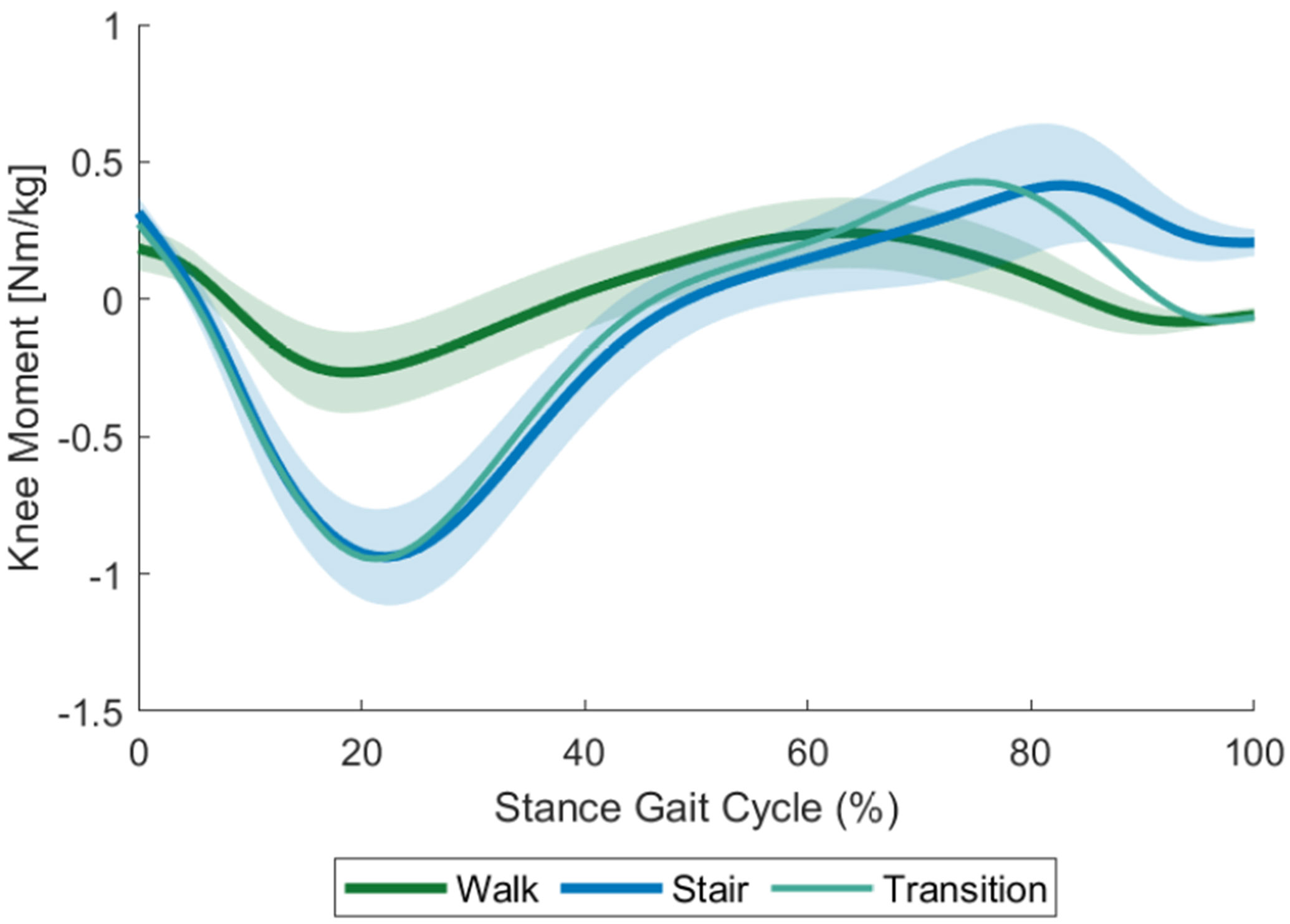
Plot of the average knee torque during steady-state stair ascent, level-ground walking, and the S2W transition at a 30° stair incline over the stance gait cycle. These averages were calculated from an AB dataset of 12 participants [38]. The transition stride knee moment, denoted in teal, closely follows the stair stride curve at the beginning of stance, gradually progressing to follow the walking stride curve by the end of stance.

During stance transition strides, knee and ankle kinetics shift gradually between steady-state motions. An example of this behavior at the knee is shown in Fig. 2. To achieve similar behavior with our HKIC approach, we model stance transition impedance within the convex hull of our W_ss_ and S_ss_ impedance models as

(3) $$\tau_{\text{st}}^{\text{tr}}=\alpha (s_{\text{st}},\chi^{\zeta_{\text{tr}}})\tau_{\text{st}}^{W}+(1-\alpha (s_{\text{st}},\chi^{\zeta_{\text{tr}}}))\tau_{\text{st}}^{S}\;\;+O(s_{\text{st}},\chi^{\zeta_{\text{tr}}})\phi (s_{\text{st}},\chi^{\zeta_{\text{tr}}}),$$

where $\tau_{\mathrm{st}}^{\mathrm{tr}}$ represents the stance joint torque during the transition stride and $\zeta_{\mathrm{tr}}\in \{W2S,S2W\}$. The interpolation coefficient, or blend factor, $\alpha (s_{\mathrm{st}},\chi^{\zeta_{\mathrm{tr}}})$ varies between 0 and 1, allowing for smooth, nonlinear transitions between steady-state impedance models as a function of stance phase. The linear torque correction $\phi (s_{\mathrm{st}},\chi^{\zeta_{\mathrm{tr}}})$ gets applied when the function $O(s_{\mathrm{st}},\chi^{\zeta_{\mathrm{tr}}})\in \{0,1\}$ determines the joint torque of the transition stride lies outside the convex hull of the corresponding torques for the two steady-state activities. The functions $\alpha (s_{\mathrm{st}},\chi^{\zeta_{\mathrm{tr}}})$ and $\phi (s_{\mathrm{st}},\chi^{\zeta_{\mathrm{tr}}})$ were formulated as polynomials reflecting stance phase and stair incline in a similar manner to the impedance coefficients of our steady-state models. Convex, quadratic-programming optimization techniques determined these functions as detailed in Section III.

During swing, we enforce desired, time-invariant joint kinematics ($\theta_{\mathrm{des}}$), known as *virtual constraints* [30]. Virtual constraints are defined for each steady-state activity based on reference AB trajectories during swing [21]. During stance transition strides, the swing controller utilizes the virtual constraints associated with the final steady-state activity. Each virtual constraint is tracked by a proportional derivative (PD) controller of the form

(4) $$\tau_{\text{sw}}=k_{p}(\theta^{\text{des}}(s_{\text{sw}},\chi^{\zeta })-\theta )-k_{d}\dot{\theta },$$

where $k_{p},k_{d}>0$ are constant proportional and derivative gains for each joint (knee or ankle). We utilize viscous damping instead of a derivative tracking term to limit vibrations that naturally arise due to the prosthesis’s minimal inherent viscous losses. While this places less emphasis on trajectory tracking, prior work has demonstrated sufficiently accurate swing-phase tracking with an appropriately chosen proportional gain [21]. Note that $\theta_{\mathrm{des}}$ corresponds to the desired kinematics, which is a key distinction from the impedance controller (2).

To smoothly transition between these stance and swing controllers, we perform time-based interpolation between $\tau_{\mathrm{st}}$ and $\tau_{\mathrm{sw}}$ outputs (calculating equations (3) and (4) in parallel) over a 200 ms window following each FS and TO event.

## III. Impedance Model for Stance Transitions

### A. Continuously-Varying Transition Model

#### 1) Model Definition:

To allow for smooth, nonlinear transitions between our W_ss_ and S_ss_ impedance models, we build a polynomial-based, piece-wise-linear model for our blend coefficient α and torque correction factor ϕ. The model is parameterized by the user’s completion fraction of stance phase $s_{\mathrm{st}}$ and task $\chi^{\zeta_{\mathrm{tr}}}$, where $\chi^{\zeta_{\mathrm{tr}}}$ is a function of stair incline angle $\gamma^{\text{stair}}\in \{19{}^{\circ},30{}^{\circ},-19{}^{\circ},-30{}^{\circ}\}$. Positive angles denote stair ascent and negative angles denote stair descent.

The model is defined by

(5) $$\alpha (s_{\text{st}},\chi^{\zeta_{\text{tr}}})=c_{\alpha }{\left(\chi^{\zeta_{\text{tr}}}\right)}^{T}{\left[s_{\text{st}}^{0}\;\;\ldots \;\;s_{\text{st}}^{d}\right]}^{T}$$

(6) $$\phi (s_{\text{st}},\chi^{\zeta_{\text{tr}}})=c_{\phi }{\left(\chi^{\zeta_{\text{tr}}}\right)}^{T}{\left[s_{\text{st}}^{0}\;\;\ldots \;\;s_{\text{st}}^{d}\right]}^{T}$$

where each polynomial coefficient selection function $c(\chi^{\zeta_{\mathrm{tr}}})\in R^{d+1\times 1}$ is the product of a constant coefficient matrix $X\in R^{d+1\times 4}$ and interpolation vector $w(\chi^{\zeta_{\mathrm{tr}}})$, ‖w‖=1. For example, $c_{\alpha }(\chi^{\zeta_{\mathrm{tr}}})$ is defined as

(7) $$c_{\alpha }(\chi^{\zeta_{\text{tr}}})=\underset{X_{\alpha }}{\underset{︸}{\left[\begin{array}{ccc} \alpha_{0,-30} & \cdots & \alpha_{0,30}\\ \vdots & \ddots & \vdots \\ \alpha_{d,-30} & \cdots & \alpha_{d,30} \end{array}\right]}}w(\chi^{\zeta_{\text{tr}}}).$$

A similar selection function is defined for the torque correction factor. To balance model flexibility and overfitting, polynomial order d=l=4 was chosen for the blend coefficient and d=m=3 for the torque correction factor.

#### 2) Model Fitting:

For our optimization, we utilize an AB dataset for steady-state stair locomotion, steady-state level-ground walking, and transitions between these two activities [38]. The dataset contains average joint kinematics and kinetics for 12 participants performing these activities on a circuit with three stair inclines. For this work, we focus on ±19° and ±30° as these incline angles (i.e., step heights) fall within the ADA-compliant range [45] and are achievable with our variable-incline staircase [21], [37]. The same dataset is used to optimize the underlying S_ss_ and W_ss_ models following the same optimization definitions and constraints detailed in [32] for walking and [21] for stairs.

The goal in fitting our transition model is to identify the optimal transition coefficient matrices $X_{\alpha }^{*}$ and $X_{\phi }^{*}$ that, utilizing our W_ss_ and S_ss_ impedance models, best reproduce the transition torque trajectories τ from the AB dataset. Similar to our optimization approach in [21], each column of our coefficient matrices only affects the impedance torque at a certain incline, meaning we can solve for each column independently. Formulating our cost function, let $x_{\alpha ,i}^{*}$ and $x_{\phi ,i}^{*}$ be the ith columns of $X_{\alpha }^{*}$ and $X_{\phi }^{*}$, respectively. Similarly, we define $\tau_{i}$, $\theta_{i}$, and ${\dot{\theta }}_{i}$ as vectors of length n of the AB transition joint torques, angles, and velocities for a given $\chi^{\zeta_{\mathrm{tr}}}$. These vectors are formed by concatenating the intra-participant average trajectories provided in the dataset. For each transition task $\chi^{\zeta_{\mathrm{tr}}}$, we wish to solve the following optimization problem:

(8) $$\{x_{\alpha ,i}^{*},x_{\phi ,i}^{*}\}=\text{arg min}\;\frac{1}{n}\;{\left\|\;\tau_{i}-{\hat{\tau }}_{i}\;\right\|}_{2}^{2},$$

where

τ^i=α(sst,i,χζtr)τst,iW+(1−α(sst,i,χζtr))τst,iS+O(sst,i,χζtr)ϕ(sst,i,χtr),O(sst,i,χζtr)={1,τi>max(τst,iW,τst,iS)1,τi<min(τst,iW,τst,iS)0,otherwise,}

A torque correction factor was not required for the knee—its introduction had negligible or negative effects on model performance due to the knee torque remaining within the convex hull of the steady-state model torques for the majority of stance. Therefore, we set ϕ=0 for the knee joint.

To reduce this optimization problem to a convex quadratic program, we can utilize the definition of our steady-state impedance model torques (3) to rewrite ${\hat{\tau }}_{i}$ as

(9) $${\hat{\tau }}_{i}=\alpha (s_{\text{st},i},\chi^{\zeta_{\text{tr}}})\tau_{i}^{\Delta }+O(s_{\text{st},i},\chi^{\zeta_{\text{tr}}})\phi (s_{\text{st},i},\chi^{\zeta_{\text{tr}}})+\tau_{\text{st},i}^{S}$$

where

$$\tau_{i}^{\Delta }=(K^{S}-K^{W})\theta_{i}+(B^{S}-B^{W}){\dot{\theta }}_{i}+(\delta^{W}-\delta^{S})$$

We then define a decision vector $x\in R^{l+m+2\times 1}=[x_{\alpha ,i}^{T},x_{\phi ,i}^{T}]$ and a matrix $E\in R^{l+m+2\times n}$ in terms of its jth column

(10) $$E[j]=[\tau_{ij}^{\Delta }s_{\text{st},ij}^{0},\cdots ,\tau_{ij}^{\Delta }s_{\text{st},ij}^{l},O(s_{\text{st},ij}^{0},\chi^{\zeta_{\text{tr}}})s_{\text{st},ij}^{0},\;\;\cdots ,{O(s_{\text{st},ij}^{m},\chi^{\zeta_{\text{tr}}})s_{\text{st},ij}^{m}]}^{T}$$

where subscript j denotes the jth component in each vector of length n. Then the cost function L(x) can be written as the quadratic function

(11) $$L(x)=\frac{1}{n}{\left(\tau_{i}-\tau_{\text{st},i}^{S}\right)}^{T}(\tau_{i}-\tau_{\text{st},i}^{S})-f^{T}x+x^{T}Hx$$

where $H=\frac{2}{n}EE^{T}$ and $f=\frac{2}{n}E(\tau_{i}-\tau_{\mathrm{st},i}^{S})$.

Because our transition impedance model is a convex sum of our steady-state impedance models, we utilize linear equality and inequality constraints to ensure that our blending coefficient 0≤α≤1. We assume that the majority, if not all, of the transition takes place during stance, using an equality constraint to ensure that α starts at 0 and ends at 1 for S2W or starts at 1 and ends at 0 for W2S. This allows for the smoothest possible transitions between our stance transition model and steady-state swing kinematic models. Our approach also assumes transitions are unidirectional, meaning that one steady-state model progresses to another as stance progresses. We therefore use an inequality constraint to ensure α decreases strictly monotonically during W2S transitions or increases strictly monotonically during S2W transitions.

Minimizing our cost function (11) subject to these constraints yields the final quadratic program (QP):

(12) $$\underset{x}{\text{minimize}}\;\;x^{T}Hx-f^{T}x,\;\text{subject to}\;\;Ax\leq b,\;A_{\text{eq}}x=b_{\text{eq}}.$$

We neglect without loss of generality the positive offset $\frac{1}{n}{\left(\tau_{i}-\tau_{\mathrm{st},i}^{S}\right)}^{T}(\tau_{i}-\tau_{\mathrm{st},i}^{S})$ originally found in our objective function L(x). This QP was solved for each stair inclination and joint using the MATLAB Optimization Toolbox (R2023b).

#### 3) Incline-Independent Transition Model:

**Fig. 3.**
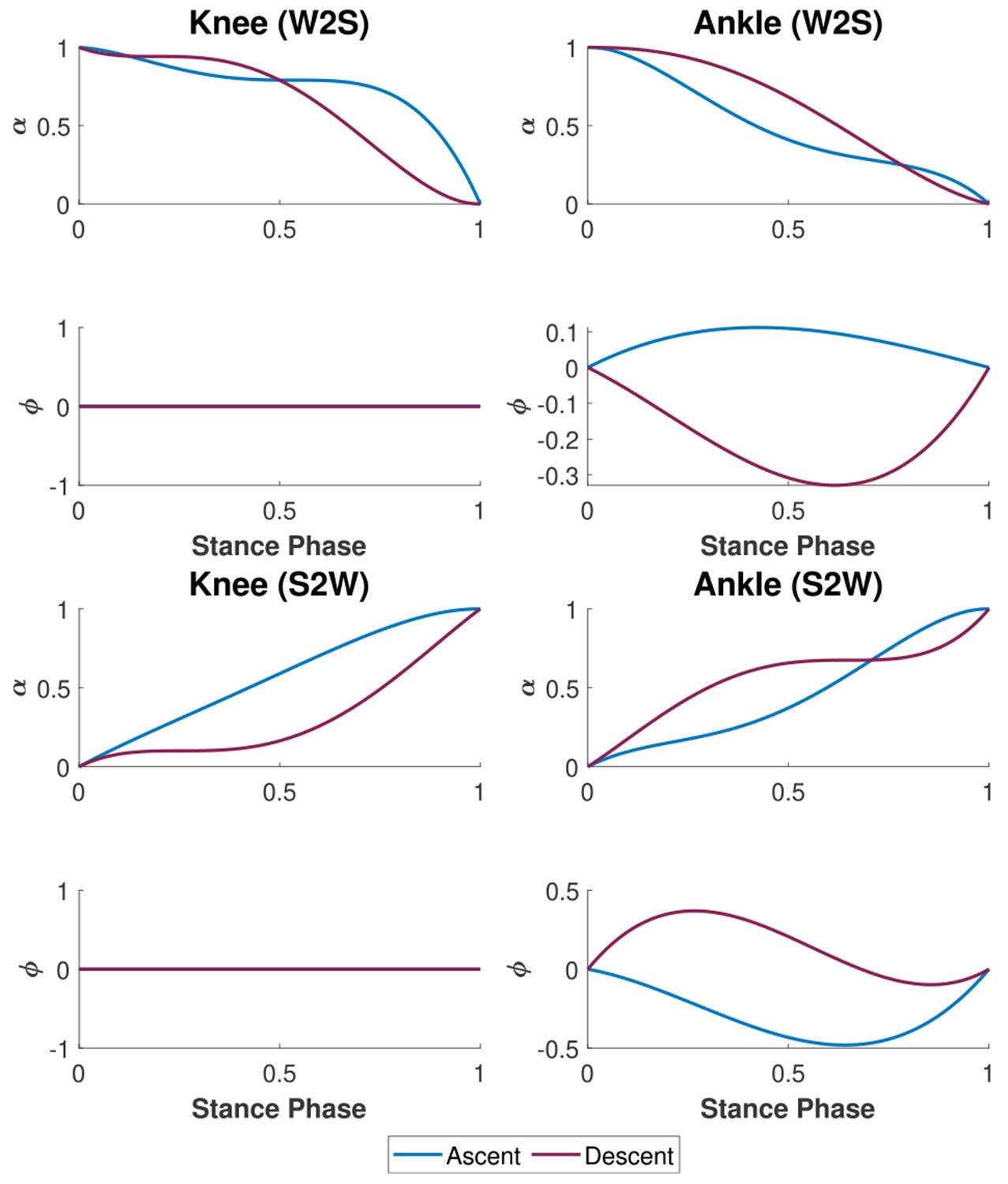
Plots of the blending coefficient α and torque correction factor ϕ for incline-independent continuous stance transition models as a function of stance phase. Ascent is denoted in blue and descent is denoted in red.

To reduce the complexity of our transition model, we modify our approach to generate a single incline-independent set of coefficients for each stance transition (W2SA, W2SD, SA2W, and SD2W). This simplification reduces the number of transition coefficient pairs used by half, from 8 total to 4. For example, we train our W2SA model utilizing the associated AB kinematic and kinetic joint data for both the 19° and 30° stair inclines. It is important to note that the stair incline must still be known when using this model due to the S_ss_ model utilized being incline-dependent (see Section V). The resulting stance transition coefficients for this model approach are shown in Fig. 3. This modeling approach is referred to as continuously-varying independent (CI) throughout the rest of this manuscript for brevity. We denote the incline-dependent version of our model as continuously-varying dependent (CD).

### B. Stance Transition Model Evaluation

**Fig. 4.**
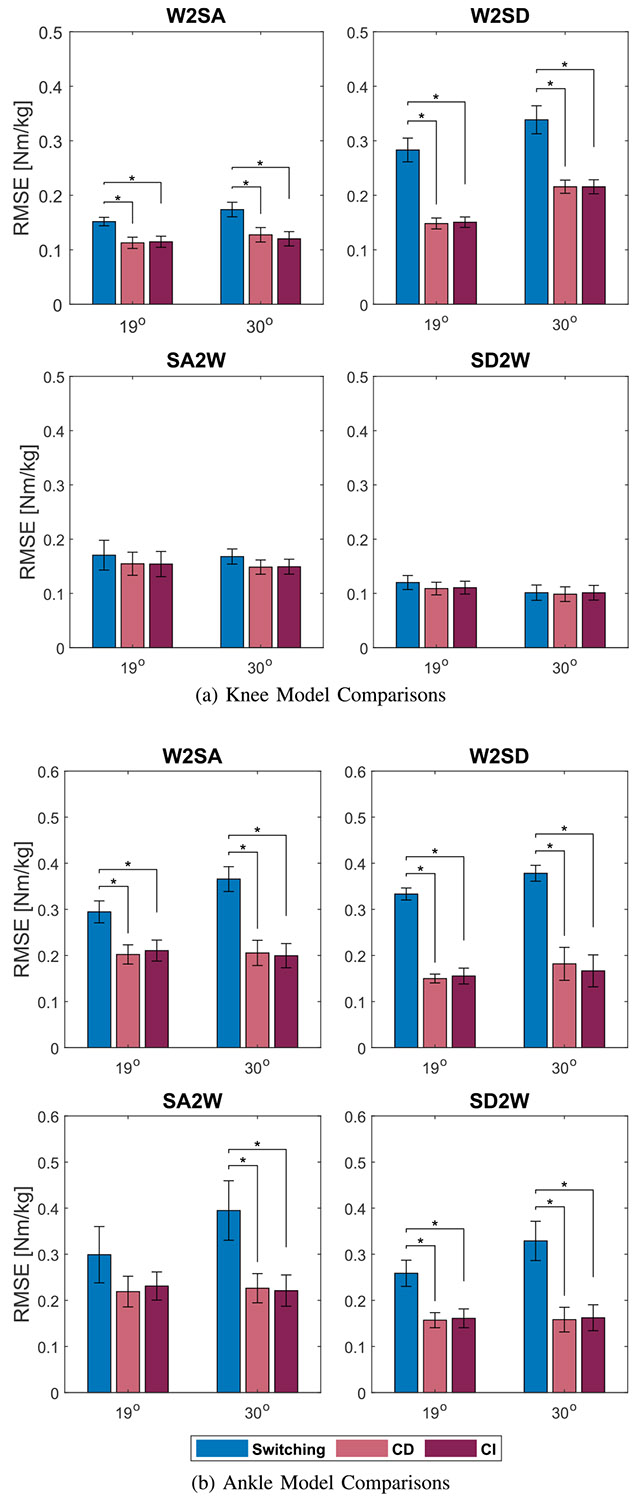
Bar plots comparing the reconstruction error in average RMSE of our continuously-varying dependent (CD), continuously-varying independent (CI), and the switching-based transition model inspired by [35] across all stance transition activities (W2SA/D and SA2W/SD2W) and stair inclines ±(19° *and* 30°). The error bars show ±1 standard error from the mean. The asterisks (*) indicate a statistically significant difference (significance level of 0.05) from the student’s t-test, comparing RMSE between models.

We compare the performance of our continuously-varying stance transition model to a simple switching-based transition controller inspired by previous impedance control methods [35] including our multi-activity classification study [42]. Specifically, the simple controller switches between our W_ss_ and S_ss_ impedance models at important events of the transition stride (which begins at prosthesis FS). For W2SA, the transition is classified at prosthesis TO of the transition stride, so the stance transition is performed entirely with the W_ss_ model. For W2SD, the transition is classified at prosthesis FS of the transition stride, so the stance transition is performed entirely with the S_ss_ model. For SD2W, the transition is classified at mid-stance of the transition stride, switching from the S_ss_ model to the W_ss_ model at this mid-stance event. Finally, for SA2W, the transition is not classified until after stance, so the S_ss_ model is chosen for the stance portion of the transition stride. We compare our continuously-varying dependent (CD), continuously-varying independent (CI), and our simple switching controller for each transition stride at each training stair incline within the AB dataset utilized for training our models. We calculate the root mean squared error (RMSE) in joint torque for each of N= 12 AB participants in the training dataset [38]. Fig. 4 shows the average RMSE of each model for each stance transition and stair incline combination at the knee (Fig. 4a) and ankle (Fig. 4b). We use a student’s t-test to determine statistical significance when comparing model RMSE, with 0.05 set as the level of significance.

At the knee, the CD and CI models perform significantly better than the corresponding switching model for each W2S transition across all inclines. However, during S2W, the switching model performs almost as well as the others. Similarly, for the ankle, the continuous transition models perform significantly better than the switching model except SA2W at the 19° incline. Across both joints and all evaluated activities and task variations, there is no significant difference between the CI and CD models. Based on this evaluation, the rest of this paper focuses on the implementation and evaluation of our CI stance transition modeling approach on a state-of-the-art powered knee-ankle prosthesis.

## IV. Amputee Experiments

### A. Methods

The incline-independent stance transition controller was implemented on the quasi-direct drive knee-ankle prosthesis from [10]. This prosthesis weighs approximately 6.5 kg (including batteries) and features high-torque, low-impedance actuators comprising the ILM 85 × 26 motor kit (RoboDrive, Seefeld, Germany) and a 22:1 planetary transmission. The motors are driven by G-SOLO Twitter R80A/80VDC drives (Elmo Motion Control, Petah Tikva, Israel). A prosthetic foot (Ottobock Lo Rider, 1E57) is mounted below a 6-axis load cell (Sunrise Instruments, Nanning, China), which mounts to the distal end of the ankle joint. The control and signal processing code is implemented on a myRIO 1900 (National Instruments, Austin, TX) mounted on the front of the prosthesis. All control code is executed at 500 Hz. The global orientation of the residual thigh is measured using a 3DM-CX5-25 IMU (LORD Microstrain, Williston, VT) affixed to the proximal end of the knee actuator. Motor positions are measured by E5, 3600 cpr optical quadrature encoders (US Digital, Vancouver, WA). Joint velocities are estimated with a first-order Butterworth filter. Due to the low inertia of the actuator design, this device can perform open-loop impedance control and high-bandwidth position control [10], allowing for the implementation of HKIC control approaches [21], [32].

Two K4 TF amputee participants were enrolled to validate the controller implementation. Participants with this high activity classification are more capable of continuously transitioning between terrains (with reduced risk of falling [4], [5]) and thus are more likely to benefit from improved transition biomechanics. Relevant participant information is detailed in Table I. The participants had at least ten hours of experience with the powered prosthesis, which was previously fit by a certified prosthetist. The experimental protocol was approved by the University of Michigan Institutional Review Board on February 27, 2023 (HUM00230065). The study involved three sessions: an acclimation session, a data collection session on a 30° incline, and a data collection session on a 19° incline. Both participants used the same control parameters generated from AB data; no participant-specific tuning was required. An overhead safety harness was offered for all procedures.

Data collection included four walk-stair transitions (W2SA, W2SD, SA2W, SD2W) at two stair inclinations on a 5-step adjustable staircase. The experimental transition circuit had a set of parallel bars leading up to the staircase and a platform following the staircase to allow for level-ground walking, to and from the staircase. Stair inclinations of 19° and 30° were chosen to match the inclinations utilized in the training dataset [38]. Due to the geometry of our adjustable staircase, these stair inclinations resulted in step heights that fall within the ADA-compliant range of 102-178 mm [45].

Ten W2S trials and ten S2W trials were performed for each stair inclination and direction. As this study focused on evaluating the mid-level control design, we assumed the stair incline and the transition stride were known by the controller (see Section V for discussion on real-time detection of transitions and stair incline). A high-level state machine, indexed by FS events, was used to switch between our HKIC controllers utilizing a pre-programmed sequence of strides for W2S (W_ss_→W2S→S_ss_) or S2W (S_ss_→S2W→W_ss_) trials as shown in Fig. 1. Each transition sequence comprised five FS events, detected with a 6-axis load cell (Sunrise Instruments, Canton, MI) mounted at the base of the device’s powered ankle joint. The W2S sequence began with one full stride of level walking, followed by an intact-leading W2S transition stride immediately before the staircase, and concluded with two subsequent stair strides. Conversely, the S2W transition sequence began with two full stair strides, followed by an intact-leading S2W transition stride at the end of the staircase, and concluded with one full stride of level walking. Upon completion of the ascent transition sequence at the top of the staircase, the participant turned around and the experimenter remotely initiated the descent transition sequence.

Participants were encouraged to maximize their loading of the device and only utilize the handrails to maintain balance. They were also encouraged to perform the tasks at a comfortable pace. 30-second breaks were given between trials, and a 1-minute break was given after the fifth trial of each set. 10-minute breaks were given after completion of all trials at a given incline to allow the experimenters to adjust the staircase to the next testing incline. Video of the participants performing each transition type is available as supplementary material.

To evaluate our continuous stance transition model, we compared kinematic and kinetic data collected by the powered prosthesis to an AB reference. The AB reference represents the inter-participant average transition stride of a given activity and incline taken from the AB dataset [38].

### B. Experimental Results

**Fig. 5.**
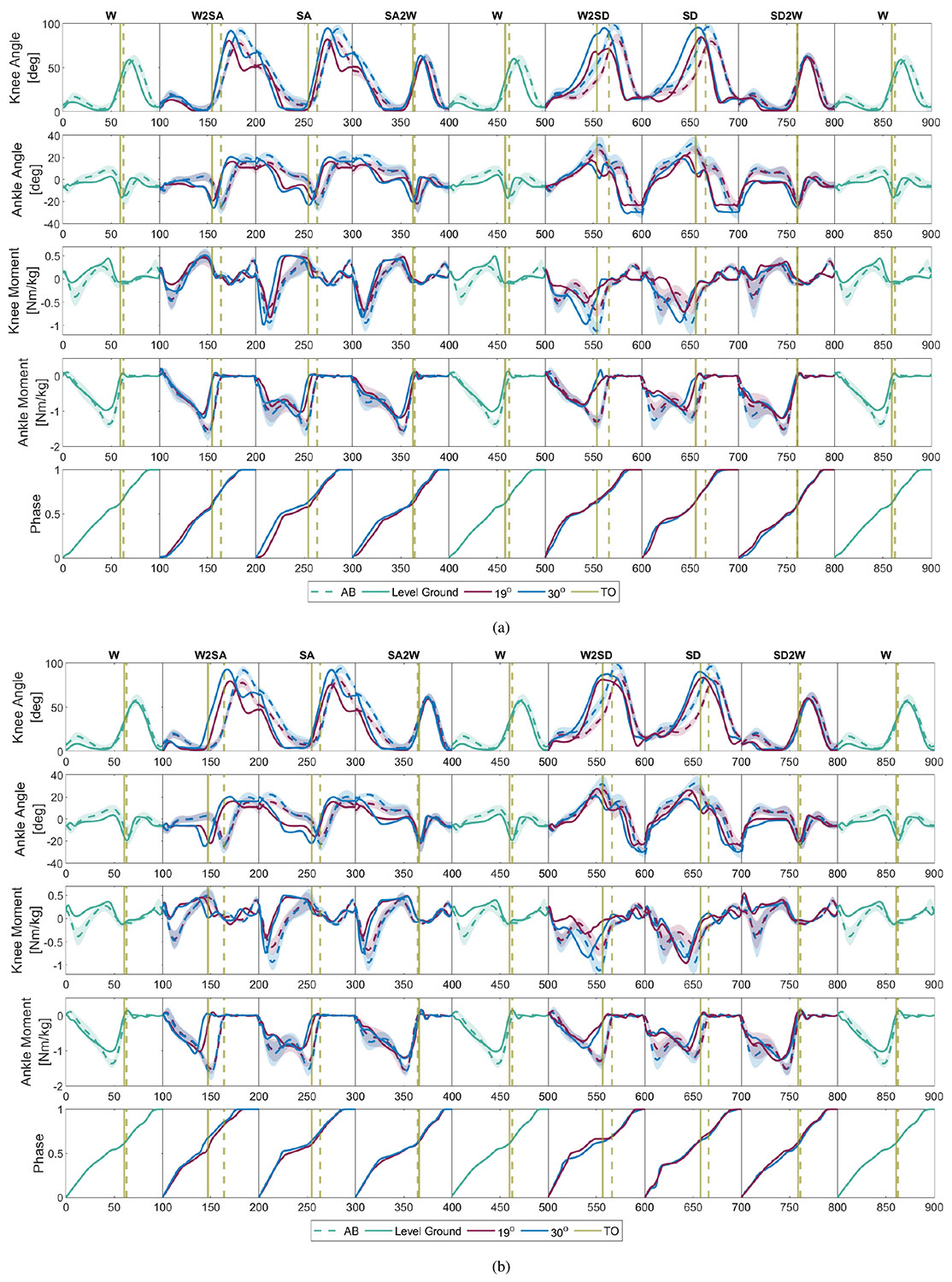
Mean prosthetic kinematics, kinetics, and phase trajectories for P1 (a) and P2 (b) over the entire testing sequence for both the 19° and 30° stair inclines. The mean and standard deviation of the AB reference for each plot are denoted with a dashed line and shaded curve, respectively. We also include the average AB and P2 TO time in normalized time for all tasks. The average is taken from the TO time measured for both stair inclines for S_ss_ and transition strides. Although S2W and W2S transition strides were collected in different trials due to the number of steps on our staircase, we chose to represent the results as one continuous sequence to show the continuity between our transition and steady-state strides.

The presented controller provided smooth, monotonic phase advancement across the transition sequences in Fig. 5, with distinctive patterns emerging during steady-state and transition strides. During stance transitions, the phase variable advanced consistently with minimal pauses. Transitions biased toward S_ss_ exhibited a reduced phase rate from mid-to-late stance, corresponding to slower thigh movement. Across all activities, phase consistently saturates towards the end of swing due to the intentional design of the phase variable. This feature allows the prosthetic joints to reach the FS configuration early, promoting user confidence for weight acceptance into the following stride (see [21], [32]). Hence, the prosthesis joint patterns slightly lead the AB References at the end of swing.

The prosthetic kinematics and kinetics in Fig. 5 confirm that stance transitions continuously blend the biomechanical patterns of the corresponding steady-state activities. Deviations from AB references largely stem from the underlying steady-state models and transition timing. For instance, the limited knee extension torque during level-ground walking extends to both the W2SA and SD2W stance transitions because the transition blend coefficient (see Fig. 3) prioritizes the walking model during high-torque portions of the transition stride. Similarly, the ankle torque did not reach biomimetic peaks during steady-state activities and consequently during the transition strides. Early TO may have contributed to reduced ankle torque generation during stair locomotion and W2S transitions, whereas W_ss_ and S2W transitions yielded TO timings closely aligned with the AB reference (Fig. 5).

The prosthetic joints generally reached normative kinematic peaks in the majority of stance transitions. Tables II and III show commonly reported features for evaluating amputee biomechanics and prosthesis performance [9], [19], emphasizing peak values rather than timings due to the altered stance-swing ratio commonly observed in amputee gait [46]. Magnitudes within a standard deviation of the AB reference were considered to be biomimetic, noting that specific AB transitions have high kinematic variance. Both participants exhibited biomimetic peak knee flexion (related to toe clearance during swing) and extension angles (related to weight acceptance during stance), which increased as expected with stair incline. An exception was observed for P2, where the prosthesis did not reach the expected knee extension angle at the beginning of the W2SD transition. This observation is consistent with the steady-state walking strides of Fig. 5b, demonstrating the direct relationship between the performance of the steady-state models and the transition controllers interpolating between them. At the ankle, both W2S and S2W stance transitions produced biomimetic magnitudes and trends in plantarflexion angle, which is critical for push-off propulsion during walking and stair ascent and for foot positioning before weight acceptance during stair descent. Ascent transitions also exhibited biomimetic peak dorsiflexion angles (important for clearing steps), but neither participant experienced biomimetic peak dorsiflexion peaks during descent transitions.

Tables II and III also show that knee kinetics were generally biomimetic across most transitions, while ankle plantarflexion torque remained consistently below normative levels. For P1, the prosthesis provided biomimetic levels of knee extension torque and work during W2SD and SA2W but did not quite reach biomimetic levels during W2SA and SD2W. P2 experienced similar biomimetic trends of increasing knee work and torque magnitude with increasing stair incline, but knee extension torque decreased for W2SA transitions. Across all cases, the ankle did not reach biomimetic levels of plantarflexion torque, despite sometimes reaching biomimetic levels of work.

## V. Discussion

**Fig. 6.**
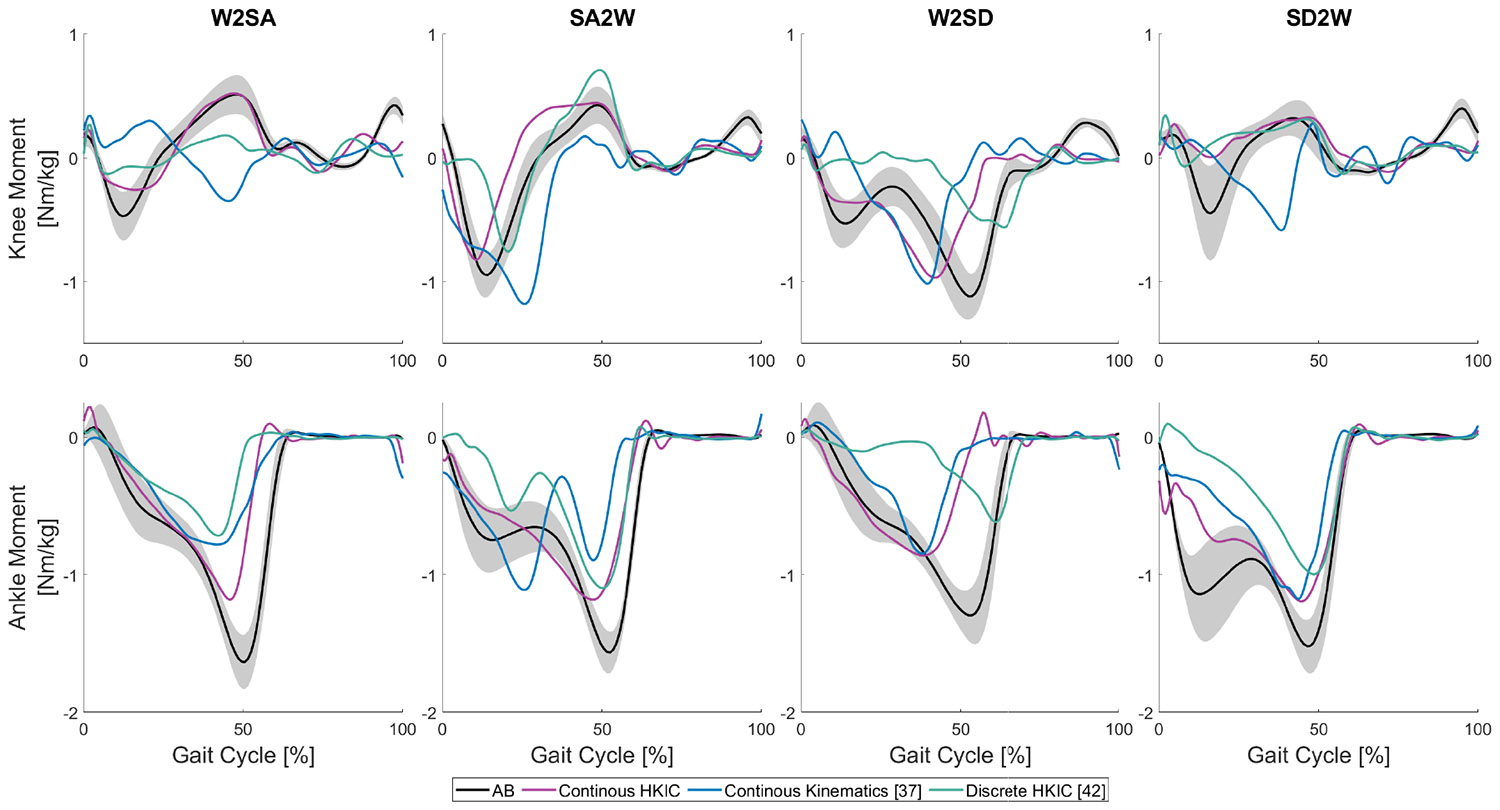
Mean prosthetic joint moments for P1 with the presented continuous HKIC transitions, continuous kinematic transitions from [37], and discrete HKIC transitions from [42]. All cases were performed at the 30° staircase configuration, and able-bodied references are given for comparison. The continuous HKIC and kinematics controllers were manually triggered as described in Section IV-A, whereas the discrete HKIC controller was automatically triggered at transition-dependent events of the transition stride (at prosthetic TO for W2SA, mid-stance for W2SD and SA2W, and FS for SD2W [42]).

In this pilot validation study, the presented prosthesis controller achieved similar knee and ankle biomechanics to AB references, with a few exceptions, during stance transitions over two stair inclines. Stance transition biomechanics were tied to the respective steady-state models, where blending progression was synchronized to the user by a common phase variable definition. This phase-based approach allowed both participants to volitionally control the timing of prosthetic joint patterns within each stride of the pre-programmed activity sequence. Compared to our prior kinematic controller for continuous walk-stair transitions [37], the presented approach offers kinetic biomimicry (Fig. 6) in addition to kinematics. Compared to prior continuous impedance controllers using discrete switching [42], the presented approach provides more normative transition biomechanics (Fig. 6). Both of these benefits improved user comfort as discussed below.

### A. Participant Feedback

We received positive feedback from our amputee participants regarding their perception of support and smoothness during transitions. P1 noted that using the powered prosthesis over the four-hour session felt like it required less effort than navigating one stair flight with their passive device following the experiment. However, P1 also noted that the additional weight of our powered device caused discomfort with their socket, which resulted in multiple adjustments and loss of suction at different points within the experiment. Implementation of this control approach on a lighter-weight device (requiring accurate position and impedance control) would likely reduce this discomfort while maintaining the noted benefits compared to P1’s passive device. P2 was not as sensitive to the weight of the device and appreciated the extra support from the knee during stair ascent and descent.

### B. Comparison to Kinematics-Based Continuous Transitions

In comparison to previous work utilizing a continuous kinematics-based controller during stance transitions [37], we demonstrated similar kinematic biomimicry across the majority of transitions with the added benefit of kinetic biomimicry during stance (Fig. 6), especially at the knee. As an example, the knee moment in W2SA appears inverted by the continuous kinematic controller but appropriately signed by the continuous HKIC controller. Additionally, the supportive knee extension torque during SD2W is substantially delayed by the continuous kinematic controller but appropriately timed by the continuous HKIC controller. These kinetic observations may explain the qualitative feedback from our repeated participants from both studies, who particularly noted the impedance controller improved their comfort during stair descent activities. However, only the kinematic-based transition controller can handle swing transitions (when the prosthesis leads the transition), as demonstrated in [37]. The combination of kinematic-based swing transitions with the presented impedance-based stance transitions would enable smoother, more natural locomotion using the HKIC-based multi-activity classification framework in [42] as discussed in Section V-D.

### C. Comparison to HKIC-Based Discrete Transitions

While the discrete HKIC controllers in [42] replicate normative joint kinetics during steady-state activities and avoid overt discontinuities during transitions (via temporal blending), Fig. 6 reveals that the discrete shifts between steady-state controllers fail to capture the nuanced kinetic profiles of AB stance transitions. In contrast, the presented continuous HKIC transitions demonstrate improved tracking of AB stance kinetics across the majority of tasks by correctly blending the two steady-state impedance models during the stance phase. Specifically, the timing and profile of the knee extension torque more closely match AB moment curves across all transitions with the exception of SD2W. In the example of the W2SA transition, the continuous controller accurately tracks the positive knee flexion torque in mid-to-late stance (approximately 30-55% stride) whereas discrete HKIC falls short. This highlights the biomechanical benefits of biomimetic blending over the entire stance period rather than a narrow time window with discrete switching. These kinetic observations align directly with qualitative feedback from our repeated participants, who reported the continuous transitions felt more natural and smooth compared to their prior experience with the discrete transitions in [42]. However, it is important to note that some of the discrete HKIC transitions in Fig. 6 may improve if timed differently than the activity classifier used in [42]. In other words, some deviations from AB biomechanics may be artifacts of the classification timing rather than limitations of the steady-state impedance models themselves.

### D. Integration With Multi-Activity HKIC Framework

In its current implementation, our stance transition control scheme is limited by the use of a predetermined activity sequence indexed by FS detection. To be used in practice, this transition control scheme must be implemented within a high-level activity classifier to automatically trigger the transition sufficiently early in the transition stride. The high-level classifier in [42] switches between steady-state HKIC controllers upon detection of a transition (at pre-determined gait phases) by linearly interpolating between the two respective controller outputs over a short time window. Instead of discretely switching to the next steady-state controller, the remaining stance period of a detected stance transition could utilize the continuous transition model to smoothly bridge the two steady-state activities. Utilizing our common phase variable across steady-state and transition activities would also simplify the multi-activity HKIC control architecture in [42] by preventing the need to simultaneously calculate multiple phase variables associated with different steady-state activities.

To maximally benefit from the presented stance transition controller, stance transitions (including ascent vs. descent) must be detected in real time near the beginning of the transition stride’s stance period. The classification framework outlined in [42] utilizes heuristics inspired by AB joint biomechanics to detect transitions before or during the transition stride with 99% accuracy. Specifically, all stance transitions can be detected within the first half of the stance period, with SD2W detected before the start of the stride. The stance transitions that are detected near mid-stance could update the transition model’s blend coefficient based on the gait phase at detection, allowing a smooth transition for the remainder of the stance period. Improvements to the classification architecture could enable even smoother continuous transitions. For example, participants who previously participated in [42] noted a smoother W2SD transition with the presented controller compared to [42], which utilized the sit-stand controller to perform the stance transition before switching to the stair descent controller. This sequence was necessary due to the classifier’s late detection of the W2SD stance transition. The speed and accuracy of high-level activity classification could potentially be improved by more sophisticated environment mapping through LIDAR or mmWave radar and/or by tracking contralateral limb behavior using a wearable IMU [47].

### E. Limitations and Other Future Work

The presented transition model assumes fixed start and end states, neglecting variations in foot placement, stride length, and locomotion speed. For instance, the relatively lower knee extension torque of P2 during stance transitions may have been caused by his faster “comfortable” pace during level-ground walking. While the time-invariant phase variable allows user-driven stride progression, the commanded joint biomechanics do not adapt in magnitude or shape (other than temporal stretching/compression). Future iterations should incorporate stride speed- and length-dependent models using expanded AB datasets or extrapolation, as current reference data [38] lack multi-speed transitions across various stair inclines.

Furthermore, the presented transition model inherits performance limitations from its underlying steady-state models. Torque deficits during W2SA and SD2W transitions—which favor walking—can be explained by suboptimal knee extension and ankle plantarflexion torques in the steady-state walking model. These underlying issues may have been caused by the optimization from [32] fitting a variety of walking inclines, declines, and speeds under the same constraints across all task variations. Task-specific constraints could potentially mitigate these limitations, though we prioritized the novel transition blending over steady-state optimization in this study.

Participant behavior and compensations adapted from passive devices may have also impacted controller performance. For example, P2 instinctively lunged into stair descent strides and associated transitions, likely causing the higher-than-expected torque magnitudes in steady-state stair descent at the 19-degree incline, lower magnitudes during W2SD, and decreased mid-to-late stance phase rates in these tasks. Furthermore, both participants exhibited premature TO across W2S and S_ss_ tasks, preventing the ankle from achieving biomimetic peak extension torques. While extended device use may help unlearn these compensatory behaviors in favor of more efficient gait biomechanics, individualizing the estimated TO phase parameters for S_ss_ could provide a better match to the user’s gait at the cost of configuration time.

Additionally, slow or low push-off torque may stem from the thigh-dependent nature of the phase variable. Slow thigh progression in mid-to-late stance—especially during stair ascent and associated transitions—directly slows phase progression and corresponding joint patterns. Although previous work [32] utilized a time-based phase state during mid-to-late stance to allow ankle progression independent of thigh motion, we intentionally retained a purely feedback-based phase definition to prioritize user volition and prevent uncontrolled, rapid stair descent. To address the corresponding tradeoff observed for stair ascent, phase variable normalization parameters could be adjusted based on locomotion speed as in [32], or phase could be calculated from the contralateral thigh during periods of slow ipsilateral thigh motion (requiring bilateral sensing) [48].

Finally, control parameters derived from population-average AB data [38] may not be optimal for individual users. While mimicking population-average AB joint biomechanics can improve gait symmetry and reduce compensatory motions [33], [34], recent studies highlight the benefits of individualizing continuous impedance models via manual tuning [49] or Principal Component Analysis [50]. Because this study was limited to two high-mobility (K4) transfemoral participants, future studies must recruit a larger cohort—including moderate-mobility (K3) individuals—to fully evaluate the clinical viability of this control approach and the potential necessity of user-specific tuning for walk-stair transitions.

## VI. Conclusion

We presented a data-driven control approach to enable continuous, biomimetic stance transitions between level-ground walking and stair ascent/descent. This controller utilized a common phase variable definition across all steady-state and transition activities. Two TF amputee participants demonstrated human-like prosthetic joint biomechanics across all four stance transitions at two stair inclines, with no individualization of control parameters. However, more participants must be evaluated before we can claim this approach generalizes across the TF amputee population, especially those with lower mobility levels. Future work can integrate this stance transition control method with the kinematic swing transition controller [37] and activity classifier [42] to potentially improve amputee biomechanics across the primary activities of daily living.

## Supplementary Material

supp1-3683020

This article has supplementary downloadable material available at https://doi.org/10.1109/TNSRE.2026.3683020, provided by the authors.

## Figures and Tables

**TABLE I:** Participant Attributes

ID	Sex	Age (yrs)	Mass (kg)	Height (m)	K-Level (K1-K4)
Pl	Male	23	83	1.75	K4
P2	Male	20	80	1.85	K4

**TABLE II:** W2S Peak Joint Biomechanics Features and Joint Work

Metric	Incline (deg)	AB	P1	P2
Knee Extension Angle (deg)	−30	7.08±3.69	4.57±1.16	1.76±1.00
	−19	6.79±3.19	3.82±0.87	1.14±0.97
	19	1.81±4.02	1.67±0.28	0.72±0.41
	30	1.98±4.29	1.68±0.20	1.73±0.48
Knee Flexion Angle (deg)	−30	98.4±5.62	96.88±3.34	92.23± 3.90
	−19	83.87±5.20	84.74±3.92	81.59±2.13
	19	77.79±5.19	81.06±3.36	80.28±2.05
	30	91.99±4.49	92.56±1.07	93.78±1.51
Ankle Plantar Flexion Angle (deg)	−30	−31.05±4.31	−32.76±0.08	−32.67±0.21
	−19	−24.91±3.93	−25.26±0.07	−25.35±0.18
	19	−22.68±6.18	−21.88±2.09	−23.42±0.83
	30	−25.72±6.51	−27.58±0.24	−26.77±0.88
Ankle Dorsiflexion Angle (deg)	−30	32.42±6.79	16.40±2.12	23.3±2.96
	−19	26.96±4.83	19.84±3.20	27.86±3.57
	19	15.27±4.17	16.22±0.11	16.14±0.14
	30	19.96±4.63	20.71±0.27	20.34±0.08
Knee Extension Torque (Nm/kg)	−30	−1.15±0.19	−1±0.29	−1.02±0.24
	−19	−0.7±0.15	−0.65±0.23	−0.41±0.22
	19	−0.44±0.19	−0.14±0.09	0.03±0.03
	30	−0.48±0.20	−0.28± 0.10	−0.01±0.03
Ankle Plantar Flexion Torque (Nm/kg)	−30	−1.33±0.06	−0.91±0.12	−0.99±0.11
	−19	−1.31±0.04	−0.89±0.15	−0.79±0.11
	19	−1.53±0.05	−1.09±0.05	−1.04±0.05
	30	−1.66±0.05	−1.22±0.06	−1.11±0.06
Knee Work (J/kg)	−30	−0.77±0.12	−0.85±0.20	−0.77±0.21
	−19	−0.37±0.07	−0.39±0.13	−0.24±0.16
	19	0.06±0.03	0.04±0.03	0.07±0.01
	30	0.07±0.04	0.02±0.01	0.09±0.01
Ankle Work (J/kg)	−30	−0.36±0.16	−0.15± 0.1	−0.24±0.13
	−19	−0.29±0.09	−0.22±0.12	−0.30±0.03
	19	0.23±0.06	0.17±0.06	0.16±0.01
	30	0.33±0.06	0.28±0.03	0.21±0.02

**TABLE III:** S2W Peak Joint Biomechanics Features and Joint Work

Metric	Incline (deg)	AB	P1	P2
Knee Extension Angle (deg)	−30	1.85±3.43	2.16±0.31	1.50±0.11
	−19	1.39±3.19	1.62±0.06	0.46± 0.20
	19	1.17±3.11	1.6±0.15	0.69±0.11
	30	1.27±3.08	2.47±0.64	1.60±0.21
Knee Flexion Angle (deg)	−30	62.89±4.99	63.94±1.19	59.78± 1
	−19	62.64± 4.50	62.62±1.83	60.37±0.99
	19	61.66±3.39	60.95±0.85	60.02±0.77
	30	66.9± 3.80	67.98±0.66	64.27±0.92
Ankle Plantar Flexion Angle (deg)	−30	−29.86±4.44	−27.34±0.86	−25.19±0.78
	−19	−24.37±5.65	−23.40±1.56	−21.70±0.73
	19	−23.46±7.33	−19.92±0.49	−20.92±0.52
	30	−24.00±6.66	−21.94±1.37	−22.40±0.44
Ankle Dorsiflexion Angle (deg)	−30	11.25±4.27	2.93±0.40	3.11± 0.5
	−19	10.04±4.02	2.72±0.51	3.50±0.37
	19	14.21±3.21	13.42±0.58	14.12±0.56
	30	22.38±1.85	20.92±1.80	19.73±0.73
Knee Extension Torque (Nm/kg)	−30	−0.5±0.32	−0.07±0.06	−0.09±0.04
	−19	−0.39±0.25	−0.04±0.02	−0.07±0.03
	19	−0.69± 0.2	−0.72±0.06	−0.43±0.07
	30	−0.96±0.18	−1.02±0.07	−0.76±0.09
Ankle Plantar Flexion Torque (Nm/kg)	−30	−1.57±0.02	−1.21±0.03	−1.25±0.02
	−19	−1.53±0.02	−1.21±0.02	−1.30±0.05
	19	−1.56±0.02	−1.20±0.04	−1.21±0.03
	30	−1.57±0.01	−1.19±0.11	−1.23±0.06
Knee Work (J/kg)	−30	0±0.04	0±0.01	−0.01±0.01
	−19	0±0.04	0.01±0.01	0.00±0.01
	19	0.18± 0.10	0.28±0.03	0.12±0.02
	30	0.37± 0.10	0.49±0.03	0.32±0.04
Ankle Work (J/kg)	−30	0.08±0.09	0.08±0.04	−0.02±0.03
	−19	0.07± 0.10	0.08±0.05	−0.01±0.04
	19	0.36±0.08	0.25±0.03	0.20±0.03
	30	0.47± 0.1	0.37±0.07	0.27±0.04
